# Programmed death-ligand 1 and mammalian target of rapamycin signaling pathway in locally advanced rectal cancer

**DOI:** 10.1007/s12672-022-00471-8

**Published:** 2022-02-14

**Authors:** Yanru Feng, Jialin Luo, Peng Liu, Yuan Zhu, Guoping Cheng, Linfeng Zheng, Luying Liu

**Affiliations:** 1 Department of Radiation Oncology, The Cancer Hospital of the University of Chinese Academy of Sciences (Zhejiang Cancer Hospital), Institute of Basic Medicine and Cancer (IBMC), Chinese Academy of Sciences, No 1, East Banshan Road, Gongshu District, Hangzhou, 310022 China; 2 Zhejiang Key Laboratory of Radiation Oncology, The Cancer Hospital of the University of Chinese Academy of Sciences (Zhejiang Cancer Hospital), Institute of Basic Medicine and Cancer (IBMC), Chinese Academy of Sciences, Hangzhou, China; 3 Department of Pathology, The Cancer Hospital of the University of Chinese Academy of Sciences (Zhejiang Cancer Hospital), Institute of Basic Medicine and Cancer (IBMC), Chinese Academy of Sciences, Hangzhou, China

**Keywords:** Rectal cancer, Programmed death-ligand 1, Chemoradiotherapy, mTOR, Survival

## Abstract

**Purpose:**

To evaluate the role of programmed death-ligand 1 (PD-L1) and mammalian target of rapamycin (mTOR) signaling pathway in locally advanced rectal cancer (LARC).

**Methods:**

Between February 2012 and February 2018, 103 patients with LARC treated by neoadjuvant chemoradiotherapy (neoCRT) and total mesorectal excision (TME) were included. PD-L1, mTOR and p-mTOR of pair-matched pre-neoCRT biopsies and post-neoCRT surgical tissue were evaluated by immunohistochemistry.

**Results:**

The mean combined positive score (CPS), tumor proportion score (TPS) and immune cell score (IC) of pre-neoCRT were 2.24 (0–70), 1.87 (0–70) and 0.67 (0–10), respectively. The mean CPS, TPS and IC of post-neoCRT were 2.19 (0–80), 1.38 (0–80) and 1.60 (0–20), respectively. Significant difference was observed in terms of IC between pre-neoCRT and post-neoCRT (p = 0.010). The 5-year disease-free survival (DFS) rate of the whole group was 62.4%. Multivariate analysis by Cox model indicated that pre-neoCRT TPS [hazard ratio (HR) 1.052, 95% confidence interval (CI) 1.020–1.086, p = 0.001] and post-neoCRT CPS (HR 0.733, 95% CI 0.555–0.967, p = 0.028) were associated with DFS. In the 89 patients without pathological complete response, p-mTOR and IC were upregulated after neoCRT.

**Conclusions:**

For patients with LARC treated by neoCRT and TME, p-mTOR and IC were upregulated after neoCRT. Pre-neoCRT TPS and post-neoCRT CPS were independent prognostic predictors of DFS.

## Introduction

Colorectal carcinoma is the second leading cause of cancer-related deaths globally, with rectal cancer accounting for approximately one third of newly diagnosed patients [1]. Since the publication of the clinical trial [2] from German Rectal Cancer Study Group, preoperative chemoradiotherapy has become a priority for patients with locally advanced rectal cancer (LARC). At present, neoadjuvant 5-fluorouracil or capecitabine based chemoradiotherapy (neoCRT) combined with total mesorectal excision (TME) is the standard treatment [3]. However, about 30% patients might develop distant metastasis after this multidisciplinary approach [4, 5]. For patients achieving clinical complete response after neoCRT, watch-and-wait is an alternative treatment strategy for preserving the rectum and avoiding surgery [6].

Over the past decade, immunotherapy has elicited promising responses in many cancers such as non-small cell lung cancer and melanoma [7]. For LARC, pathological complete response (pCR) rates of 30% and 60% were achieved in microsatellite stable (MSS) and microsatellite instability-high (MSI-H) patients treated by immunotherapy (Nivolumab, anti-programmed death 1 monoclonal antibody) combined with neoCRT [8]. However, the role of programmed death-ligand 1 (PD-L1) in rectal cancer is controversial [9]. Combined positive score (CPS) determined by PD-L1 staining has been identified for predicting anti-PD1 efficacy in head and neck squamous cell carcinoma and gastric cancer [10, 11]. To date, the data of PD-L1 scores including tumor proportion score (TPS), combined positive score (CPS), and immune cell score (IC) for LARC is limited [12, 13].

Mammalian target of rapamycin (mTOR) signaling pathway regulates various biological functions including cell growth, metabolism and immune response [14]. In addition, it is often abnormally activated and regulates the differentiation and function of T cells in tumors [15]. In LARC after neoCRT, p-mTOR was significantly overexpressed and high p-mTOR and p-S6 levels correlated with the development of distant metastasis [16]. By cBioPortal and Weighted Gene Co-expression Network Analysis of The Cancer Genome Atlas and Genotype-Tissue Expression databases, mTOR signaling pathway was correlated with PD-L1 dysregulation in gastrointestinal cancers [17]. The aim of this study was to evaluate the role of PD-L1 and mTOR signaling pathway for LARC patients treated by neoCRT.

## Methods

### Patients

The present study was a single-center retrospective and observational study. The study design adhered to the Strengthening the Reporting of Observational Studies in Epidemiology guidelines [17]. Between February 2012 and February 2018, 108 patients with LARC were treated by neoCRT and TME in our center. Of these, 5 patients were not included in the present study for limited rectal specimens. Concurrent capecitabine based pelvic intensity-modulated radiotherapy to the primary tumor and to the mesorectal, presacral, and internal iliac lymph nodes was given with 45–50.4 Gy in 25–28 fractions at 1.8–2.0 Gy per daily fraction. Two patients did not receive concurrent chemotherapy for old age. TME was performed 6 to 8 weeks after completing neoCRT. Adjuvant capecitabine or capecitabine plus oxaliplatin was given at the discretion of the attending physicians. All patients were staged based on the 8th edition of the American Joint Committee on Cancer (AJCC) staging system.

### Immunohistochemistry

Immunohistochemistry (IHC) of 5 μm sections from formalin-fixed paraffin embedded tissue of 103 pair-matched pre-neoCRT biopsies and post-neoCRT surgical tissue was performed with mTOR (clone 7C10; Cell Signaling Technology [CST], Japan; 1:100 dilution), phosphorylated mTOR (p-mTOR, Ser2448, clone 49F9; CST; 1:100 dilution), and PD-L1 (Clone MIH1, eBioscience, San Diego, California, USA; dilution, 1:50).

Immunostaining for mTOR and p-mTOR was assessed by a semiquantitative histology scoring method. The staining intensity was scored as 0 (no), 1 (weak), 2 (moderate) and 3 (strong), respectively. The percentage of stained cells was scored as 0 (0–10%), 1 (11–25%), 2 (26–50%) and 3 (51–100%). The overall score was the product of the intensity and percentage scores, ranging from 0 to 9 [16]. Tumour cell PD-L1 expression was evaluated based on immunostaining in the cytoplasm and membrane of tumour cells. The cytoplasmic PD-L1 staining intensity of tumor cell was scored as 0 (absent), 1 (weak), 2 (moderate) and 3 (strong), respectively. The tumor cell membrane PD-L1 staining intensity was scored as 0 (absent) and 1 (present), respectively. The PD-L1 expression score of tumor cells was determined by adding the cytoplasmic and membrane scores, ranging from 0 to 4 [11]. Each PD-L1 staining section was assessed according to the TPS (PD-L1-stained tumor cells/total number of viable tumor cells×100), CPS (PD-L1-stained tumor cells and immune cells/total number of viable tumor cells×100), and IC (PD-L1-stained immune cells/total number of viable tumor cells×100) [12].

### Statistical analysis

The Statistical Package for Social Sciences, version 22.0 (IBM, Armonk, NY, USA) was used for statistical analysis. The Kaplan–Meier estimation method was used to assess the disease-free survival (DFS) and overall survival (OS). OS was defined as the time from the first day of neoCRT to death for any reason or the day of last follow-up. DFS was determined from the first day of neoCRT to the date of tumor recurrence or distant metastasis. Cox models were used to assess prognostic factors using backward to eliminate the insignificant explanatory variables. Age and sex were covariates in all tests and other factors included distance from anal verge, cT classification, cN classification, concurrent chemotherapy, adjuvant chemotherapy, pCR, pre-neoCRT mTOR expression score, pre-neoCRT p-mTOR expression score, microsatellite instability status, pre-neoCRT TPS, pre-neoCRT CPS, pre-neoCRT IC, post-neoCRT CPS, and post-neoCRT IC. Pre-neoCRT mTOR expression score, pre-neoCRT p-mTOR expression score, pre-neoCRT TPS, pre-neoCRT CPS, pre-neoCRT IC, post-neoCRT CPS, and post-neoCRT IC were defined as continuous variables for multivariate analysis. All statistical tests were two sided, and p < 0.05 was considered to be statistically significant.

## Results

### Clinical characteristics and tumor cell PD-L1 expression

**Fig. 1:**
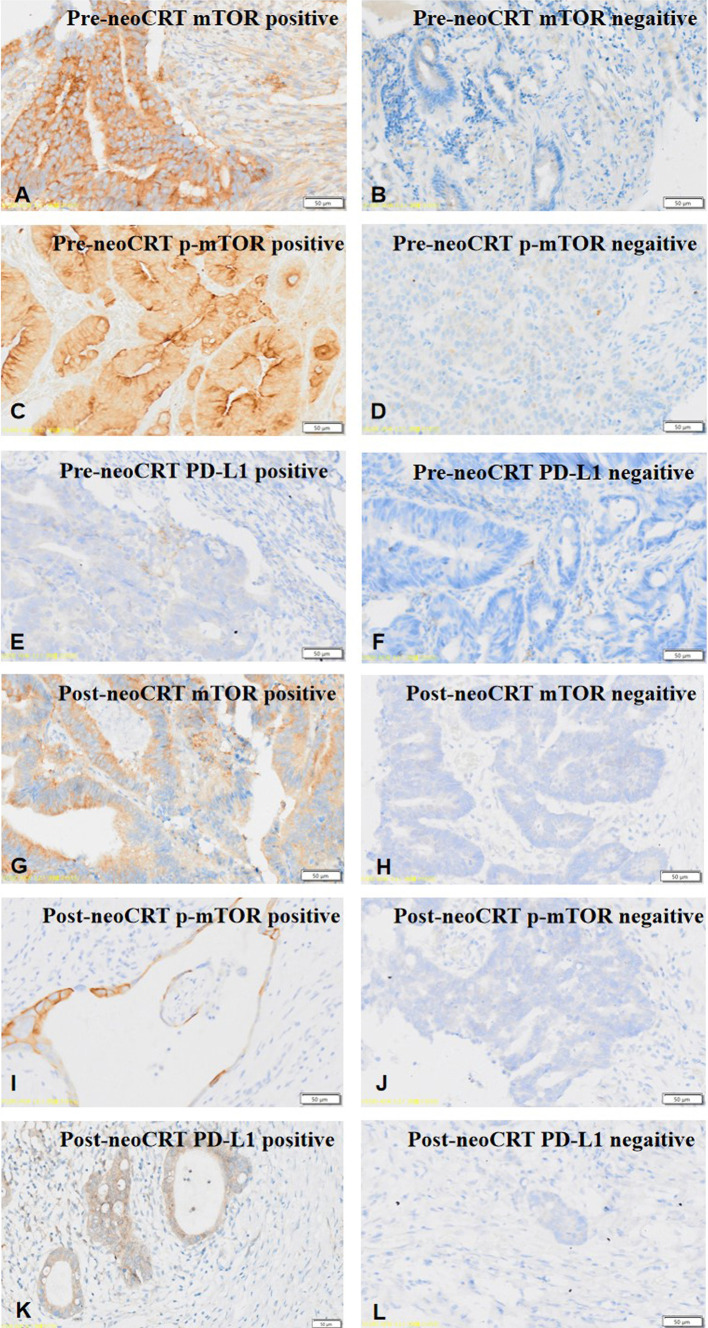
Representative immunohistochemistry staining of mTOR (**A**, **B**, **G**, **H**), p-mTOR (**C**, **D**, **I**, **J**) and programmed death ligand 1 (PD-L1) (**E**, **F**, **K**, **L**) of the pre-neoCRT tissues and post-neoCRT biopsies in locally advanced rectal cancer

The details of clinical characteristics and PD-L1 expression are listed in Table 1. The mean tumor cell PD-L1 expression scores of the post-neoCRT tissues and pre-neoCRT biopsies were 0.19 (0–4) and 0.21 (0–3), respectively. Based on the pre-neoCRT tumor cell PD-L1 expression, less patients with T4b classification and lower mTOR expression were observed in the score 0 group. The microsatellite instability status was correlated with the tumour PD-L1 expression score in pre-neoCRT biopsies. Representative immunohistochemistry staining of mTOR (Fig. 1A, B, G, H), p-mTOR (Fig. 1C, D, I, J) and programmed death ligand 1 (PD-L1) (Fig. 1E, F, K, L) of the pre-neoCRT tissues and post-neoCRT biopsies in locally advanced rectal cancer.

**Table 1:** Clinical characteristics

Characteristic	Total cases, n (%)	Tumour PD-L1 expression score (pre-neoCRT)			Tumour PD-L1 expression score (post-neoCRT)			
		0, n (%)	1–3, n (%)	p value	0, n (%)	1–4, n (%)	p value*	pCR, n (%)
Sex								
Male	75 (72.8)	66 (74.2)	9 (64.3)	0.440	61 (74.4)	4 (57.1)	0.381	10 (71.4)
Female	28 (27.2)	23 (25.8)	5 (35.7)		21 (25.6)	3 (42.9)		4 (28.6)
Age (years)								
Median	58	59	54.5	0.580	55.5	61	0.629	60
Range	28–73	28–73	29–64		28–73	29–68		45–72
Distance from anal verge (cm)								
≤ 5 cm	63 (61.2)	55 (61.8)	8 (57.1)	0.740	49 (59.8)	4 (57.1)	> 0.999	10 (71.4)
> 5 cm	40 (38.8)	34 (38.2)	6 (42.9)		33 (40.2)	3 (42.9)		4 (28.6)
cT classification								
T3	61 (59.2)	55 (61.8)	6 (42.9)	0.035	50 (61.0)	3 (42.9)	0.305	8 (57.1)
T4a	19 (18.4)	18 (20.2)	1 (7.1)		16 (19.5)	1 (14.3)		2 (14.3)
T4b	23 (22.3)	16 (18.0)	7 (50.0)		16 (19.5)	3 (42.9)		4 (28.6)
cN classification								
N0	15 (14.6)	15 (16.9)	0 (0)	0.129	9 (11.0)	1 (14.3)	0.434	5 (35.7)
N1	50 (48.5)	44 (49.4)	6 (42.9)		42 (51.2)	2 (28.6)		6 (42.9)
N2	38 (36.9)	30 (33.7)	8 (57.1)		31 (37.8)	4 (57.1)		3 (21.4)
Concurrent chemotherapy								
Yes	101 (98.1)	87 (97.8)	14 (100.0)	> 0.999	80 (97.6)	7 (100.0)	> 0.999	14 (100.0)
No	2 (1.9)	2 (2.2)	0 (0)		2 (2.4)	0 (0)		0 (0)
Adjuvant chemotherapy								
Yes	81 (78.6)	68 (76.4)	13 (92.9)	0.292	67 (81.7)	6 (85.7)	> 0.999	8 (57.1)
No	22 (21.4)	21 (23.6)	1 (7.1)		15 (18.3)	1 (14.3)		6 (42.9)
pCR								
Yes	14 (13.6)	12 (13.5)	2 (14.3)	> 0.999	-	-	-	-
No	89 (86.4)	77 (86.5)	12 (85.7)		-	-		-
Pre-neoadjuvant chemoradiotherapy mTOR expression score								
Median	2	2	6	< 0.001	2	6	0.141	1.5
Range	0–9	0–9	0–9		0–9	0–9		0–9
Pre-neoadjuvant chemoradiotherapy p-mTOR expression score								
Median	0	0	0	0.339	0	0	0.753	0
Range	0–9	0–9	0–9		0–9	0–6		0–6
Post-neoadjuvant chemoradiotherapy mTOR expression score (n = 89)								
Median	3	3	3	0.883	3	3	0.653	-
Range	0–6	0–6	0–6		0–6	0–6		-
Post-neoadjuvant chemoradiotherapy p-mTOR expression score (n = 89)								
Median	0	0	0	0.482	0	0	0.736	-
Range	0–9	0–9	0–9		0–9	0–9		-
Microsatellite instability status								
Proficient	31 (30.1)	25 (28.1)	6 (42.9)	0.015	27 (32.9)	3 (42.9)	0.303	1 (7.1)
Deficient	6 (5.8)	3 (3.4)	3 (21.4)		4 (4.9)	1 (14.3)		1 (7.1)
NA	66 (64.1)	61 (68.5)	5 (35.7)		51 (62.2)	3 (42.9)		12 (85.7)

neoCRT, neoadjuvant chemoradiotherapy; pCR, pathologic complete remission; mTOR, mammalian target of rapamycin; PD-L1, programmed death-ligand 1; NA, not available

*The contrast test did not include the “pCR” group

### PD‑L1 scores and survival

The mean CPS, TPS and IC of pre-neoCRT were 2.24 (0–70), 1.87 (0–70) and 0.67 (0–10), respectively. The mean CPS, TPS and IC of post-neoCRT were 2.19 (0–80), 1.38 (0–80) and 1.60 (0–20), respectively. Elevated IC was observed after neoadjuvant chemoradiotherapy (p = 0.010). With median follow-up time of 64.5 (3.5–109.8) months, the 5-year OS and DFS rates were 73.4% and 62.4%, respectively. There were only 4 patients with pre-neoCRT TPS ≥10 and 5 patients with post-neoCRT CPS ≥10. The 5-year DFS rates of patients with post-neoCRT CPS ≥1 and <1 were 69.6% and 56.1%, respectively (p = 0.254). The 5-year DFS rates of patients with pre-neoCRT TPS ≥1 and <1 were 52.2% and 64.0%, respectively (p = 0.269). Multivariate analysis by Cox model indicated that pre-neoCRT TPS [hazard ratio (HR) 1.052, 95% confidence interval (CI) 1.020-1.086, p = 0.001] and post-neoCRT CPS (HR 0.733, 95% CI 0.555–0.967, p = 0.028) were associated with DFS.

### mTOR signaling pathway and PD‑L1 expression for pCR and non-pCR

Fourteen patients achieved pCR after neoCRT. No significant differences were observed in terms of mTOR signaling pathway and PD‑L1 expression between the pCR and non-pCR groups. The details of mTOR signaling pathway and PD‑L1 expression between the pCR and non-pCR groups were shown in Table 2.

**Table 2:** mTOR signaling pathway and PD‑L1 expression for pCR

	Non-pCR	pCR	p value
Pre–neoCRT			
Mean PD–L1 scores	0.21 (0–3)	0.21 (0–2)	0.996
Mean IC	0.71 (0–10)	0.43 (0–5)	0.588
Mean CPS	2.54 (0–70)	0.36 (0–2)	0.422
Mean TPS	2.15 (0–70)	0.14 (0–1)	0.462
Mean mTOR	2.94 (0–9)	2.79 (0–9)	0.858
Mean p–mTOR	1.29 (0–9)	1.14 (0–6)	0.833
Post–neoCRT			
Mean IC	1.75 (0–20)	0.64 (0–2)	0.266
Mean CPS	2.49 (0–80)	0.29 (0–1)	0.384

neoCRT, neoadjuvant chemoradiotherapy; pCR, pathologic complete remission; mTOR, mammalian target of rapamycin; PD-L1, programmed death-ligand 1; CPS, combined positive score; TPS, tumor proportion score; IC, immune cell score

**Fig. 2:**
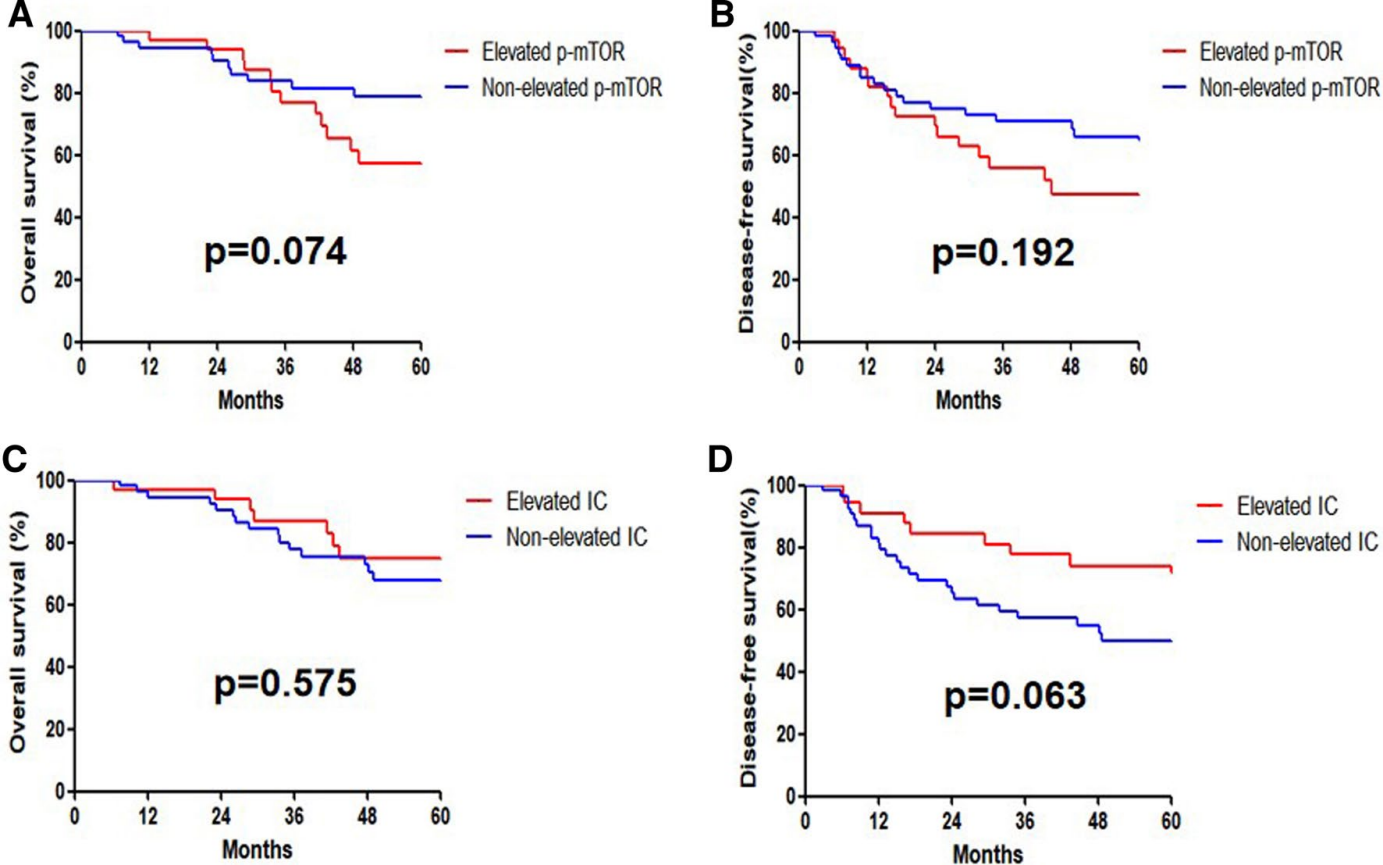
Kaplan–Meier curves of overall survival (OS) and disease-free survival (DFS) in the subgroups of locally advanced rectal cancer patients with non-pCR after neoadjuvant chemoradiotherapy (**A** OS of patients with Elevated p-mTOR and Non-elevated p-mTOR; **B** DFS of patients with Elevated p-mTOR and Non-elevated p-mTOR; **C** OS of patients with Elevated IC and Non-elevated IC; **D** DFS of patients with Elevated IC and Non-elevated IC)

In the 89 patients with non-pCR, p-mTOR and IC were upregulated after neoCRT (Table 3). Compared with the expression of pre-neoCRT, the 5-year OS rates of patients with elevated and non-elevated p-mTOR after neoCRT were 57.3% and 78.9%, respectively (p = 0.074) (Fig. 2 A). The 5-year DFS rates of patients with elevated and non-elevated p-mTOR after neoCRT were 47.3% and 65.9%, respectively (p = 0.192) (Fig. 2B). The 5-year OS rates of patients with elevated and non-elevated IC after neoCRT were 74.8% and 67.6%, respectively (p = 0.575) (Fig. 2C). The 5-year DFS rates of patients with elevated and non-elevated IC after neoCRT were 73.8% and 49.4%, respectively (p = 0.063) (Fig. 2D).

**Table 3:** mTOR signaling pathway and PD‑L1 expression for non-pCR

	Pre-neoCRT	Post-neoCRT	p value
Mean PD–L1 scores	0.21 (0–3)	0.19 (0–4)	0.801
Mean IC	0.71 (0–10)	1.75 (0–20)	0.012
Mean CPS	2.54 (0–70)	2.49 (0–80)	0.974
Mean TPS	2.15 (0–70)	1.38 (0–80)	0.569
Mean mTOR	2.94 (0–9)	2.92 (0–6)	0.975
Mean p-mTOR	1.29 (0–9)	2.56 (0–9)	0.003

neoCRT, neoadjuvant chemoradiotherapy; pCR, pathologic complete remission; mTOR, mammalian target of rapamycin; PD-L1, programmed death-ligand 1; CPS, combined positive score; TPS, tumor proportion score; IC, immune cell score

## Discussion

In the present study, the relationship between PD-L1 and mTOR signaling pathway and clinical outcomes of LARC after neoCRT was assessed. Our data indicated that the pre-neoCRT TPS and post-neoCRT CPS were independent prognostic predictors of DFS and the expression levels of p-mTOR and IC were elevated in patients with non-pCR after neoCRT.

KEYNOTE-048 study indicated that immunotherapy alone was associated with increased OS in the recurrent or metastatic squamous cell carcinoma of the head and neck patients with CPS ≥ 1 or ≥ 20 [19]. For advanced gastric/gastroesophageal junction (G/GEJ) cancer, CPS ≥10 was observed as a biomarker for predicting the efficiency of immunotherapy based on KEYNOTE-059, KEYNOTE-061 and KEYNOTE-062 studies [11]. In the present study, post-neoCRT CPS was associated with DFS. The role of CPS predicting the efficiency of immunotherapy for the locally advanced rectal cancer should be elucidated in the future. In the study including 72 patients with rectal cancer after neoCRT, low PD-L1 TPS prior to neoCRT was associated with inferior survival (HR 0.29, 95% CI: 0.11–0.76, p = 0.01) [13], which was similar to our study. Colorectal cancers with microsatellite instability-high have favorable response to the anti-PD-1 immunotherapy [20]. In the present study, the microsatellite instability status was correlated with the tumour PD-L1 expression score in pre-neoCRT biopsies. The possible mechanism resulted from the increased tumors immunogenicity and lymphocytic infltration [21].

In colorectal cancer, p-mTOR overexpression was significantly associated with the occurrence of distal and lymph node metastasis [22]. However, in another study including 1800 colorectal cancers, no significant association between p-mTOR expression and patients’ gender, tumor stage, tumor grade or nodal status was observed. In a multivariate analysis including including pT, pN, tumor grade, tumor localization and p-mTOR expression, p-mTOR could not be confirmed to be a biomarker for prognosis (p = 0.8879) [23]. Recently, Shiratori et al. reported 98 rectal cancer patients after neoCRT and 80 colorectal cancer patients without neoCRT. Compared with colorectal cancer patients without neoCRT, post-neoCRT p-mTOR was significantly overexpressed in the rectal cancer after neoCRT. High post-neoCRT p-mTOR was associated with distant metastasis in rectal cancer patients after neoCRT [16]. In the present study, pre-neoCRT p-mTOR was not associated with the prognosis and p-mTOR expression was upregulated after neoCRT. Marginal siginificant difference was observed in terms of OS between patients with elevated and non-elevated p-mTOR after neoCRT.

mTOR signaling pathway plays an important role in the cancer immunity and many other biological funcions. However, the currently published studies on mTOR signaling pathway and PD-L1 have generated conflicting results probably because of using the different anti-PD-L1 antibodies and concentrations of mTOR inhibitor [24]. Gastrointestinal cancer data from TCGA and GTEX databases indicated that PD-L1 affected the mTOR signaling pathway [17]. In the present study, lower pre-neoCRT mTOR expression was observed in the tumor cell PD-L1 score 0 group. Of interest, p-mTOR and IC upregulated after neoCRT.

In 1975, rapamycin was first isolated. However, clinical outcomes of rapamycin as an anticancer agent were disappointing [25]. Recently, the combination of mTOR inhibition with PD-1 or PD-L1 blockade immunotherapy has been revived [14]. In the renal cell carcinoma xenografted mouse model, the combination of mTOR inhibitors with PD-L1 blockade could enhance the therapeutic efficacy of tumor suppression [26]. Clinical trials indicated mTOR inhibitors (rapamycin and everolimus) combined with chemoradiotherapy or radiotherapy for LARC was feasible and the possible mechanism of PI3K/AKT/mTOR inhibitor in enhancing radiotherapy resulted from the decreasing DNA repair [27–31]. In the present study, p-mTOR and IC upregulated after neoCRT in LARC. Further study of combing chemoradiotherapy, immunotherapy with mTOR inhibitors for LARC is warranted.

This retrospective analysis has several limitations. Firstly, high surgical volume is associated with clinical outcomes for rectal cancer [32]. The inclusion period of locally advanced rectal cancer patients in our study was 6 years (2012–2018), which was comparable to other studies [12, 13]. Secondly, all the included LARC patients were treated at a single center and no patients were treated by PD-1/PD-L1 antibodies, leaving the prediction value of PD-L1 scores uncertain at this moment. Cytokines contribute to colorectal tumorigenesis and the combination of cytokines with the anti-PD-L1/PD-1 therapy could enhance antitumor immune responses [33, 34]. The relationship among mTOR signaling pathway, PD-L1 and cytokines in rectal cancer should be elucidated in the future. At last, only one PD-L1 antibody was adopted and the different PD-L1 antibodies might influence the results of PD-L1 scores [35].

## Conclusions

For patients with LARC treated by neoCRT, p-mTOR and IC were upregulated after neoCRT. Pre-neoCRT TPS and post-neoCRT CPS were independent prognostic predictors of DFS.

## Data Availability

Our data can not be made publicly available for ethical reasons. Data are from the present study whose authors may be contacted at liuly@zjcc.org.cn or Department of Radiation Oncology, Zhejiang Cancer Hospital, Hangzhou, China.
